# Temperature Controlled Radiofrequency Ablation

**Published:** 2002-07-01

**Authors:** Olaf J Eick

**Affiliations:** Sr. Scientist, Bakken Research Center Maastricht, the Netherlands

Since its introduction in 1987, radiofrequency (RF) ablation has developed to become the treatment of choice for symptoms caused by atrio ventricular (AV) reentrant tachycardia, isthmus related atrial flutter, AV-nodal reentrant tachycardia and to some extent also for certain types of ventricular tachycardias. The introduction of new cardiac activation mapping systems has further contributed to the successful and safe application of RF ablation for various tachyarrhythmias.

## Historical Background

In 1979, the first catheter ablation was performed coincidentally when Fontaine et al. observed a complete AV block in a patient undergoing defibrillation while one defibrillator electrode was in electrical contact with a catheter electrode positioned at the bundle of His [1]. In the early 80's high voltage direct current ablation was further developed by Gallagher and Scheinman and used as a therapeutic approach to treat supraventricular tachycardias [2,3]. Although highly effective, direct current ablation was accompanied by severe complications such as cardiac tamponade, hypotension following shock delivery and the induction of ventricular arrhythmias, which stimulated the search for alternative energy sources. Huang et al. investigated in 1987 experimentally the use of radiofrequency energy for catheter ablation [4], followed by the first AV-node ablation by Budde et al [5] and the interruption of an accessory pathway by Borggreffe et al [6].

The advantages of radiofrequency energy as compared to direct current are the generation of well circumscribed lesions without stimulation or sensory effects. In order to more appropriate titrate radiofrequency power, catheter tip temperature monitoring was investigated experimentally in 1989 by Hindricks et al. and Haines et al [7]. In 1992 Langberg et al. published their observations of using temperature monitoring with a thermistor embedded in the distal electrode during catheter ablation of accessory pathways in 20 patients [8]. As a result of a constant power delivery, the tip temperature increased gradually to reach a steady state within a few seconds. Neither the applied power nor the electrogram characteristics at the ablation site (amplitude, signal stability) could predict tip temperature. Lower temperatures were found when transient blocks had been induced (50 ± 8° C) than when permanent blocks resulted in accessory pathway ablation (62 ± 15° C). Complications were not reported. Consequently, a closed loop temperature control system was introduced to regulate the applied power such that a preset target temperature could be reached and maintained during radiofrequency delivery. Calkins et al reported in 1994 a multicenter experience with 270 consecutive patients undergoing catheter ablation of supraventricular arrhythmias [9]. Compared to the power control mode, application of radiofrequency energy delivered using the temperature control mode was associated with a threefold reduction in the incidence of developing a coagulum and a fivefold reduction in the incidence of automatic power shutdown due to an impedance rise or an electrode temperature of greater than 100° C. Whether the target temperature was reached or not was related to the ablation site, with ablation of the atrioventricular junction resulting in the highest temperatures and ablation of AVNRT the lowest. In addition, the temperature of the applications of radiofrequency energy that resulted in successful ablation was not significantly different from the temperature of those that failed.

## Tissue Heating

In RF ablation, the heating of cardiac tissue is mainly resistive. RF current is applied to the tissue via a metal electrode at the tip of the catheter, with a large skin electrode serving as indifferent electrode. The current density patterns in the tissue are determined by electrode size and geometry, electrode contact and local tissue properties. Also, of course, the current density will be proportional to the current (I) delivered by the RF generator, which, for constant resistance (R) of the electrode-tissue volume conductor is proportional to the square root of the RF power (P = I2 R).

Current flow through a resistive medium causes heating, which is proportional to the square of the current or, locally, to the square of the local current density. The temperature increase will locally be proportional to the energy applied per second (local power), and increases inversely proportional to the heat capacity of the local medium. In addition, when temperature differences between adjacent areas develop because of differences in local current density or local heat capacity, heat will conduct from "hotter" to "colder" areas, causing the temperature of the former to decrease and that of the latter to increase. Additionally, heat loss to the blood pool at the surface and to intramyocardial vessels determine the temperature profile within the tissue.

## Temperature Distribution

The heating occurs especially in the proximity of the active electrode due to its relatively small surface area causing locally high current density as compared to the site of the indifferent electrode. Typically, living tissue will be permanently destroyed at temperatures of approximately 45° to 50° C sustained for several seconds [10].

**Figure 1:**
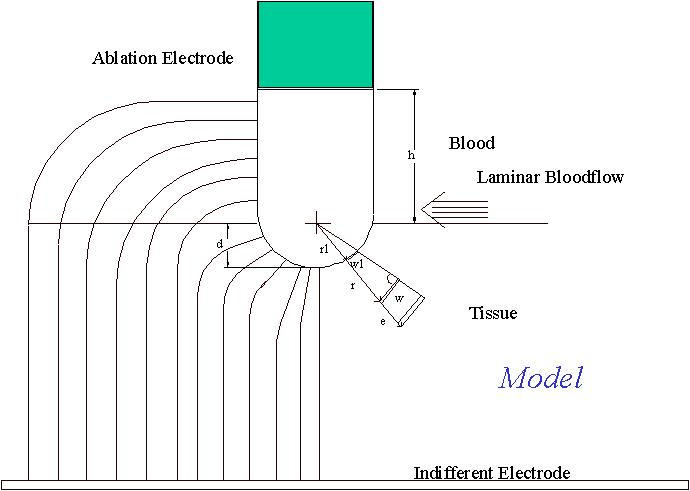
Model for radiofrequency ablation simulating a 7 F electrode, 4 mm in length and positioned perpendicularly to the tissue. Heat convection within the tissue and heat loss due to convective cooling were considered and temperatures over time and distance were calculated by solving the appropriate heat equations.

**Figure 2:**
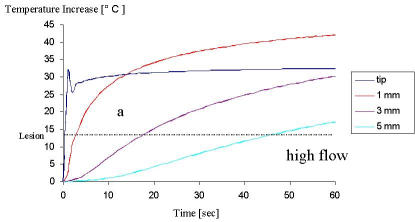

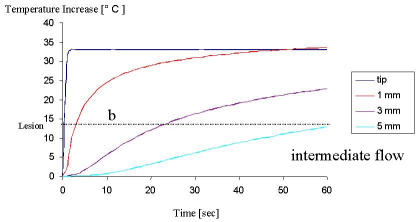

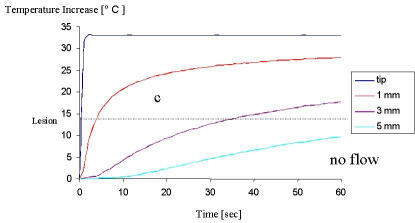
Temperature behavior over time at the electrode tip and 1, 3 and 5mm deep within the tissue for different flow conditions (a, high flow; b, intermediate flow and c, no flow) with a target temperature of 70° C, i.e. a temperature increase of 33° C and power limit of 50 W.

The tissue surface is cooled by the blood flow and thus the highest temperature during radiofrequency delivery occurs slightly below the surface. In a numerical model, the temperature distribution during radiofrequency energy was simulated [11]. The model described a 7 F electrode, 4 mm in length positioned perpendicularly to the tissue, and considered heat convection within the tissue and heat loss due to convective cooling (Figure 1). Figure 2a, b, and c illustrate the temperature behavior at the electrode tip and 1, 3 and 5mm deep within the tissue for different flow conditions with a target temperature of 70° C, i.e. a temperature increase of 33° C, and a power limit of 50 W. The catheter tip temperature rises rapidly at the beginning. The temperatures within the tissue rise more slowly than the tip temperature because it takes time for heat to transfer from the hotter rim around the electrode into the tissue. After 10 s the temperature at 1 mm becomes higher than the electrode temperature for high flow conditions (Figure 2a) and after 50 s with intermediate flow (Figure 2b) whereas the temperature is always highest at the tissue surface if there is no convective cooling (Figure 2c). In addition, the temperatures at a certain depth are higher under high flow condition resulting in deeper lesions as compared to those under moderate flow and no flow, respectively.

This temperature behavior was confirmed experimentally by Cao et al. who performed temperature measurements in vitro with multiple thermocouple needles at different depths and recorded the temperature behavior during ablation [12]. The catheter tip temperature rose rapidly at the beginning. It approached the target temperature (60° C) in about 2 to 3 s. Then the RF generator adjusted the delivered power to maintain the target temperature. When the ablation stopped, tip temperature dropped rapidly due to convective cooling by the blood flow. The temperatures measured within the tissue (0.8 mm, 2.0 mm, and 2.9 mm) rose more slowly and after 30 s the temperature at 0.8 mm depth became higher than the electrode temperature.

The heating of cardiac tissue by radiofrequency current thus depends on the current density distribution in the tissue, the tissue's heat capacity and heat conduction properties and heat convection.

## Catheter Tip Temperature

The catheter tip temperature in turn depends on the tissue temperature but also on convective cooling by the surrounding blood, and the tissue contact of the ablation electrode [13-15]. In addition, the measured catheter tip temperature depends on the electrode material with its heat capacity and the type and location of the temperature sensor. There are mainly 2 different types of temperature sensors, i.e. a thermocouple and a thermistor. The thermistor requires a driving current and the electrical resistance changes as the temperature of the electric conductor changes. More frequently used are thermocouples based on the so called 'Seebeck effect'. When 2 different metals are connected (sensing junction) a voltage can be measured at the reference junction that is proportional to the temperature difference between the 2 junctions. For temperature measurements during radiofrequency ablation typically type T thermocouples are being used that consist out of copper and constantan wires and are incorporated in the center of the ablation electrode.

## Relation between Tip temperature, Tissue Temperature and Lesion Size

Radiofrequency current heats cardiac tissue and in turn the catheter electrode is being heated. Consequently, the catheter tip temperature is always lower - or ideally equal - than the superficial tissue temperature.

The catheter tip temperature, which is measured and used to control the radiofrequency power output, can be significantly lower than the tissue temperature [16,17]. Kongsgaard et al. evaluated the differences between tip temperature and tissue temperature in vitro for different catheter electrode dimensions and flow rates during temperature controlled radiofrequency delivery [16]. With a thermosensor placed approximately 1 mm beneath a 4 mm catheter, the temperature within the tissue was on average 42 ± 6° C higher than at the electrode tip after 30 seconds radiofrequency delivery with a preset target temperature of 70° C.

Only under standardized and stable experimental conditions of flow and electrode-tissue contact the tip temperature correlates to lesion size because increasing target temperature then increases power consumption and in turn increases lesion size [7,14].

During temperature controlled radiofrequency ablation tip temperature, tissue temperature and lesion size are affected by electrode-tissue contact and cooling effects by the blood flow. Petersen et al. investigated the effect of convective cooling on lesion dimensions [18]. In vivo, 2 different application sites in the left ventricle of pigs were ablated with a target temperature of 80° C using a 4 mm tip catheter. With higher convective cooling at septal sites, the delivered power to reach target temperature was higher resulting in deeper and greater lesions than at apical sites. The applied power was positively related to lesion volume (r= 0.66) whereas the measured tip temperature was not (r= -0.49).

It is important to understand that radiofrequency power delivered to the tissue determines lesion size and that the catheter tip temperature is poorly correlated to lesion size in vivo. With good contact between catheter tip and tissue and low cooling of the catheter tip the target temperature can be reached with little power resulting in fairly small lesions although a high tip temperature is being measured. In contrast, a low tip temperature can be due to a high level of convective cooling resulting in high power consumption to reach target temperature yielding relatively large lesions. This is best illustrated with active cooling of the catheter tip using irrigation during radiofrequency energy delivery. The tip temperature is usually below 40° C but that allows the application of high power output for longer durations.

Nakagawa et al. compared the lesion dimensions produced with saline-irrigated electrodes with those produced with non-irrigated electrodes in a canine thigh muscle preparation [19]. With saline irrigation at 20 ml/min high radiofrequency voltage (66V) could be applied over 60 s resulting in lesion volumes of 700 ± 217 mm3, whereas constant radiofrequency delivery was terminated prematurely due to an impedance rise at tip temperatures of nearly 100° C resulting in lesions of only 135 ± 33 mm3 . In the temperature controlled mode with a target temperature of 80° C lesions with a volume of 275 ± 55 mm3 were produced. These results were further confirmed in an animal study performed by Skrumeda et al. who reported significantly larger lesions using irrigation during constant power delivery as compared to non-irrigated ablations either in temperature or power controlled mode [20].

With high irrigation flow rates catheter tip temperature is not representative for tissue temperature and therefore feedback cannot be used to control power output. However, Petersen et al. demonstrated the feasibility of tip temperature controlled irrigated radiofrequency delivery with low irrigation flow rates yielding larger lesions compared to non-irrigated ablations  [21].

A similar effect can be observed with large tip electrodes. With large electrode tips a larger area of the electrode tip is exposed to the blood flow than with standard tip lengths resulting in higher power consumption and lower tip temperatures yielding larger lesions [22].

In addition to cooling effects, the electrode tissue-contact affects the catheter tip temperature and the difference between tip temperature and tissue temperature. Avitall et al. evaluated the influence of electrode tissue contact on temperature rise and lesion size under stable flow conditions in an in vivo animal model during constant power delivery [23]. They demonstrated a higher temperature rise within the first 10 seconds and an increase in lesion volume with improving electrode-tissue contact.

With poor contact between electrode tip and tissue less electrode material is in contact with the tissue and heating of the tip by the tissue occurs at a smaller rate resulting in relatively low tip temperatures. As a consequence, the RF generator increases power output in order to reach target temperature.

If the electrode-tissue interface temperature increases above the boiling point due to inadequately high power delivery, any liquids under the electrode will boil and vapor will form causing a popping sound. This is often associated with sudden impedance rise and catheter dislodgment and may cause significant tissue damage [17,24]. As evaporation may also occur intramurally, a gas bubble may develop within the tissue under the electrode. Continuous application of radiofrequency energy will cause the bubble to expand and its pressure to increase, which may lead to eruption of the gas bubble through the weakest path, leaving behind a gaping hole. The release of the gas bubble is associated with a popping sound and likely with tearing of cardiac tissue [25]. Furthermore, if blood is between the electrode and the tissue during delivery of radiofrequency energy, a rapid increase in temperature may result in coagulum formation on the metallic surface of the electrode. Coagulum formation, in turn, causes a rapid increase in voltage across the rising impedance, which may result in arcing and tissue charring.

## Outlook

Since catheter tip temperature is affected by cooling effects and electrode-tissue contact it would be ideal to monitor in real time the tissue temperature during radiofrequency energy delivery. Recently, it was shown in an in vitro study that the lesion size becomes less dependent from cooling effects and electrode-tissue contact if the radiofrequency power is controlled by the tissue temperature instead of the catheter tip temperature [26]. An extractable needle that measures the tissue temperature at a certain depth may allow tissue temperature feedback to the RF generator. Erdogan et al. suggested to measure an electrochemical potential at the ablation electrode that detects the release of free radicals during lesion generation [27]. In vitro they could demonstrate that this potential correlates to lesion size. In addition, new technologies may be implemented in the future including infrared sensors and ultrasound transducers to monitor tissue temperatures during radiofrequency delivery.

## Conclusion

Monitoring catheter tip temperature and closed loop control of power output are useful to avoid excessive heating at the tissue surface, that may result in coagulum formation, and to accomplish effective heating at the target area. However, the catheter tip temperature is affected by cooling effects and electrode-tissue contact and thus poorly correlated to lesion size. New means should be developed to assess tissue temperatures during radiofrequency delivery to achieve predictable and reproducible lesions.
